# Nuclear Proteome Map of Mouse Heart Chambers

**DOI:** 10.1016/j.mcpro.2026.101585

**Published:** 2026-05-14

**Authors:** Seyed Sadegh Eslami, Alin Rai, Haoyun Fang, Anita Thomas, Jonathon Cross, Daniel Donner, David W. Greening

**Affiliations:** 1 Baker Heart and Diabetes Institute, Melbourne, Victoria, Australia; 2 Baker Department of Cardiovascular Research, Translation and Implementation La Trobe University, Melbourne, Victoria, Australia; 3 Baker Department of Cardiometabolic Health, University of Melbourne, Melbourne, Victoria, Australia

**Keywords:** heart, cardiac anatomy, subcellular, nuclear, proteome remodeling

## Abstract

Heart specialization involves nuclear programs; however, chamber-specific regulation of the nuclear proteome landscape remains unknown. In this study, we isolated the nucleus from four major anatomical regions of healthy mouse heart (fresh) and employed quantitative mass spectrometry-based proteomics to construct a comprehensive nuclear proteome landscape of left ventricle (LV, 2403 proteins), right ventricle (RV, 2242 proteins), left atrium (LA, 2368 proteins), and right atrium (RA, 1816 proteins). This led to the discovery of nuclear regional proteome signatures (ventricular signature, 297 proteins; atrial signature, 183 proteins) associated with oxidative metabolism and redox regulation, ferroptosis, extracellular-matrix remodeling, SUMO- and stress-responsive control and transcriptional regulation. Chamber-level analyses further identify distinct nuclear features in LV (120 proteins), LA (188 proteins), and RA (72 proteins). In addition, we defined conserved core nuclear proteome (230 proteins) shared across all anatomical regions, enriched for transcription-regulator complexes, nucleolar/ribosome-associated, RNA-processing, and chromatin-organization components. Within this core network, we report 78 transcription factors/co-factors and select nuclear, chromatin and RNA export-associated proteins, including 29 specific factors (*e.g.*, Alpk3, Rbm14, Arglu1, Hmgb1, Myef2, Sf1) associated with the heart. Regionally, we verified spatial localization in heart of H2ac21 and Sun2 in LA and Ptbp2 in LV by immunofluorescence. This study provides insights into the chamber-resolved view of the nuclear proteome in the heart, establishes a framework for linking nuclear proteomic signatures to atrial and ventricular biology, unique features of the heart nuclear proteome landscape relative to other organs, and a baseline for studying nuclear remodeling in cardiac pathophysiology.

## Highlights

• Heart chamber-specific regulation of the nuclear proteome landscape remains unknown.
• Nuclear fractionation and MS-based proteomics provide comprehensive maps of cardiac protein networks.
• Discovered nuclear regional proteome signatures (L/R ventricle, L/R atrium), including transcription factor/co-factor networks.
• Identify high-confidence conserved heart nuclear proteome landscape (230 proteins).
• New insights into the resolution of heart organization of cytoplasm and nuclear proteome.
• Validate spatial localization of distinct nuclear markers in heart.

## Graphical Abstract

**Figure:**
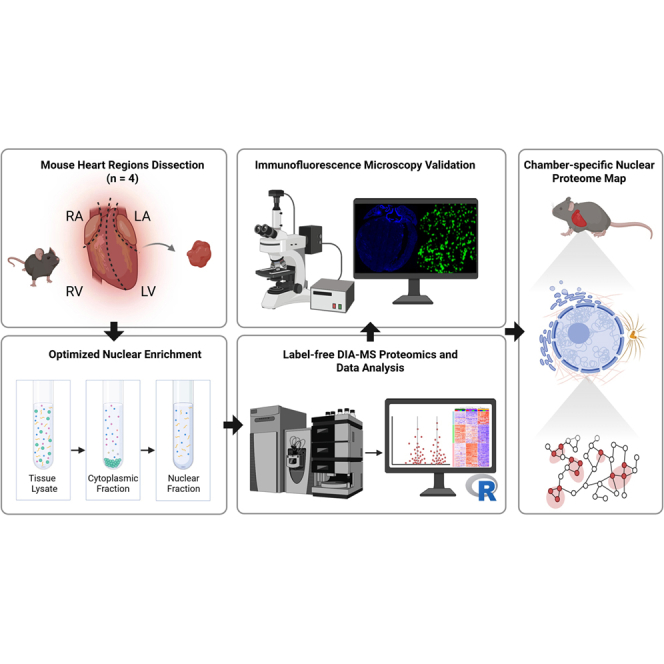

## In Brief

Heart specialization involves nuclear programs; however, chamber-specific regulation of the nuclear proteome landscape remains unknown. Here, we provide a comprehensive landscape of the heart nuclear landscape, an improved strategy to fractionate, define, and validate the nuclear (and cytoplasmic) proteome of the mouse heart in its native state. The nucleus was isolated from four major anatomical regions of a fresh, healthy mouse heart and employed quantitative mass spectrometry-based proteomics to construct a comprehensive nuclear proteome landscape and validated using spatial localisation microscopy. This led to the discovery of nuclear regional proteome signatures, the resolution of heart-select nuclear proteome, and a diversity in transcription factor networks in the heart. This study provides a resource of the differential and conserved proteome landscape of the nuclear proteome of the mouse heart and a baseline for studying nuclear remodelling in cardiac physiopathology.

The heart is a highly organized and metabolically active organ that sustains systemic circulation through rhythmic contraction and relaxation. Its four chambers, the left and right ventricles (LV, RV) and atria (LA, RA), operate under distinct mechanical and metabolic loads; the atria receive the blood from the circulation, whereas the ventricles pump blood out of the heart into the circulation, driving chamber-specific specialization that is essential for synchronized function (1, 2, 3). This diversity depends on the precise coordination of proteins governing contraction, energy metabolism, and signal transduction (4, 5). The nucleus is the center of this regulatory network, acting as a dynamic control hub that integrates biochemical and biomechanical cues to direct gene expression and chromatin organization (6, 7). Mechanical strain, calcium oscillations, and oxidative stress are transmitted to the nucleus through mechano-responsive complexes such as the LINC complex and transcriptional regulators, including MRTF-A and YAP/TAZ (8, 9, 10, 11). Together, these nuclear signaling pathways orchestrate transcriptional programs vital for maintaining myocardial performance and adaptive remodeling, while their dysregulation contributes to fibrosis, hypertrophy, and heart failure (12, 13, 14, 15).

Nuclear-cytoplasmic localization is a crucial mechanism controlling transcription factors and chromatin regulators (16), with bulk (global) proteome analyses challenged in resolving subcellular compartmentalization (17). As a result, the cardiac nuclear proteome remains poorly defined. Nuclear proteins are typically low-abundance, often chromatin-bound, and the myocardium’s dense contractile architecture further limits their recovery and detection (18, 19, 20); concomitant transcriptional and chromatin-associated regulators are consistently underrepresented in conventional cardiac proteomic datasets (19, 21). Improving nuclear coverage, therefore, requires targeted enrichment strategies, such as subcellular fractionation, to increase nuclear specificity, enhance proteome depth, and minimize cytoplasmic carryover (19, 22, 23).

Large-scale cardiac proteomic studies, including the multi-chamber cardiac atlas revealed extensive proteome depth and chamber-specific metabolic and structural features (24). Extending these efforts to the nuclear compartment requires nucleus-enriched proteomics, given that such global proteome and anatomical combined datasets do not explicitly capture subcellular compartmentalization. An extensive subcellular proteomics workflow for analyses of nuclear proteomes have been applied to other organs, including brain, liver, and kidney (23). Here, we optimized a nuclear enrichment workflow for fresh mouse heart tissue regions and quantified over 4000 proteins per region, including more than 2900 nuclear-enriched proteins and a diverse transcription factor and co-factor network. Functional analyses revealed region-dominant nuclear programs spanning oxidative metabolism, ferroptosis, extracellular-matrix remodeling, and transcriptional regulation. Comparative analysis reveals unique features of the heart nuclear proteome landscape relative to other organ nuclear proteomes (23). With stringent inclusion criteria, we further defined a conserved nuclear core shared across cardiac regions, comprising 230 proteins mapped to canonical nuclear compartments, such as chromatin, nucleoplasm, and nucleolus. This comprehensive nuclear map provides a reference for linking cardiac regional biology to nuclear protein programs and provides a foundation for future efforts to target nuclear signaling in cardiovascular disease.

## Experimental Procedures

### Experimental Design and Statistical Rationale

Nuclear-enriched proteomic data were generated from freshly dissected mouse heart regions: left ventricle including septum (LV), right ventricle (RV), left atrium (LA), and right atrium (RA), collected from four biological replicates (representing independent hearts). Cytoplasmic and nuclear fractions were prepared alongside a homogenized heart-region “global” sample for label-free quantitative MS acquisition (fractions: 32 MS files; global: 15 MS files). For Western blot validation, seven frozen whole hearts were used (three processed as described with the manufacturer’s kit and four with the optimized protocol). For immunofluorescence (IF) imaging, three formalin-fixed paraffin-embedded (FFPE) hearts were examined.

Replicate variation, data structure, and the influence of missing-value imputation were assessed using coefficient of variation (CV) analyses and principal component analysis (PCA, prcomp algorithm). Differential protein abundance across fractions was evaluated using linear modeling in limma with empirical Bayes moderation. False discovery rates (FDR) were controlled using the Benjamini–Hochberg method. Proteins with FDR-adjusted *p*-values <0.01 were considered significantly different across fractions; for nuclear-fraction regional comparisons, an FDR threshold of <0.05 was applied.

### Mouse Heart Tissue Sourcing

All mouse experiments and tissue collection were approved by the Alfred Research Alliance Animal Ethics Committee (approval number P2580). Wild-type female C57BL/6 mice (12–14 weeks old) were sourced from AMREP AS Pty Ltd (VIC). Following euthanasia, hearts were isolated immediately, rinsed in ice-cold PBS, dissected into defined chambers, and snap-frozen on dry ice. For immunofluorescence, hearts were isolated, washed in PBS, and transferred into 10% neutral-buffered formalin (NBF) for 24 h.

### Heart Region Subcellular Fractionation

Subcellular fractionation was performed using the NE-PER Nuclear and Cytoplasmic Extraction Reagents (Thermo Fisher Scientific, 78833) with workflow optimizations for cardiac tissue. Fresh dissected heart regions (4–20 mg) were transferred into pre-chilled 1.5-mL tubes containing precooled 1-mm stainless steel beads (Next Advance) and homogenized in CER I supplemented with Halt Protease and Phosphatase Inhibitor Cocktail using a pre-chilled Bullet Blender at setting six for 15 s (two cycles) followed by setting eight for 15 s. Reagent volumes were scaled to tissue mass according to the manufacturer’s recommendations, with a ∼20% increase in buffer-to-tissue ratio applied for LA and RA samples to ensure proper homogenization. All steps performed at 4 °C.

Homogenates were clarified at 100*g* for 5 min in a fixed-angle rotor centrifuge (Eppendorf 5804R; TLA-55 rotor). A portion of this clarified homogenate was collected as the Global fraction. CER II reagent was added to the remaining supernatant and centrifuged at 1,000*g* for 10 min to minimize cytoplasmic carryover. The resulting supernatant was collected as the cytoplasmic fraction. The nuclear pellet was washed once with ice-cold PBS and centrifuged at 15,000*g* for 5 min at 4 °C. The pellet was then resuspended in Nuclear Extraction Reagent (NER) and incubated on ice for 40 min with intermittent vortexing to facilitate nuclear protein solubilization. A further centrifugation was performed at 16,000*g* (4 °C) to obtain the nuclear extract, which was collected and stored at −80 °C. Protein concentrations were quantified using the microBCA assay (Thermo Fisher, 23235).

### Western Blot Analysis

Western blotting was performed on LV fractions (10 μg protein) as previously described (25). Primary antibodies used were rabbit anti-Fumarase (FH1; CST 4567), rabbit anti-Calnexin (CANX; Abcam 10286), rabbit anti-Histone H3 (CST 4499), mouse anti-Lamin A/C (LMNA; CST 4567), and rabbit anti-GAPDH (CST 97166). Secondary antibodies included IRDye 800 goat anti-mouse IgG (LI-COR 926-32210) and IRDye 680 goat anti-rabbit IgG (LI-COR 926-68071).

### Immunofluorescence Staining, Slide Scanning, and Image Analysis

Mouse hearts were fixed overnight in 10% neutral-buffered formalin, dehydrated, paraffin-embedded, and sectioned at 5 μm onto Superfrost Plus slides. Sections were baked at 65 °C, deparaffinized in xylene, rehydrated through graded ethanol, and subjected to heat-induced epitope retrieval for 30 min at 98 °C in either Dako S1699 target retrieval solution (SUN2 and PTBP2) or Tris–EDTA, pH 9.0 (Histone H2A). After washing in Dako wash buffer (K8000), sections were blocked in Dako protein block (X0909; 30–60 min, RT).

Slides were incubated for 60 min, RT with rabbit anti-PTBP2 (Invitrogen PA5-101026; 1:50), rabbit anti-SUN2 (Invitrogen PA5-95741; 1:50), or rabbit anti-Histone H2A (Abcam ab177308; 1:1000) diluted in Dako antibody diluent (S0809), followed by donkey anti-rabbit Alexa Fluor 488 (Jackson 711-545-152; 1:1000, 30 min). After further washes, membranes were labeled with WGA–Alexa Fluor 555 (1:500, 30 min). Nuclei were counterstained with DAPI (15 min, RT), autofluorescence was reduced with brief Sudan Black B treatment (0.3% in 70% ethanol), and slides were mounted in ProLong Gold Antifade (Thermo Fisher).

Whole-slide fluorescence images were acquired on an Olympus VS200 widefield slide scanner using fixed exposure and gain settings across samples. Quantification was performed in QuPath v0.5.1. Nuclei were segmented on the DAPI channel, and mean nuclear Alexa Fluor 488 intensity was measured within DAPI masks, using the WGA signal to exclude membrane/cytoplasmic regions. For each chamber, three ROIs per section were analyzed in three hearts (n = 3), and regional values were averaged for comparison with DIA-MS–derived nuclear protein abundances. Proteomics measurements were obtained from an independent cohort (n = 4); concordance was evaluated based on regional dominance patterns rather than one-to-one fold changes.

### Proteomics Sample Preparation

Samples from Global, Cytoplasmic, and Nuclear fractions (2 μg protein each) were prepared for proteomics using a bead-based SP3 workflow (19, 26). Proteins were lysed in SDS/HEPES buffer (50 mM HEPES, pH 8.0) supplemented with HALT protease/phosphatase inhibitors, then denatured in 2% SDS and reduced with 10 mM DTT followed by alkylation with 20 mM iodoacetamide (IAA) in the dark. Excess IAA was quenched by adding DTT. Proteins were subsequently captured on Sera-Mag magnetic beads (SP3), bound in the presence of ethanol, and washed repeatedly with 80% ethanol to remove detergents and other contaminants. Bead-bound proteins were then digested overnight at 37 °C using sequence-grade trypsin and Lys-C (enzyme-to-protein ratios of 1:50 and 1:100, respectively). Resulting peptide digests were collected, acidified to 1% formic acid, and dried by vacuum centrifugation. Peptides were reconstituted in 0.07% TFA and quantified using a fluorometric peptide assay (Thermo Fisher Scientific, 23,290) prior to LC–MS/MS analysis.

### Liquid Chromatography and DIA-MS

LC–MS/MS analysis was performed on a Q Exactive HF-X Orbitrap mass spectrometer coupled online to an UltiMate NCS-3500RS nano-UHPLC system, operated in positive-ion data-independent acquisition (DIA) mode as described previously (26, 27). For each injection, 300 ng of peptides were first loaded onto a C18 trapping column (PepMap100, 3 μm, 100 Å) in 0.1% formic acid, then separated on a 250-mm C18 analytical column (1.9 μm, 75 μm ID) maintained at 55 °C. Peptides were eluted using a linear gradient of 2 to 28% acetonitrile containing 0.1% formic acid over 45 min, followed by a ramp from 28 to 80% acetonitrile between 45 and 47 min, for a total run time of 56 min at a flow rate of 300 nL min^-1^. MS1 scans were acquired over an m/z range of 350 to 1100 at 60,000 resolution with an AGC target of 3 × 10^6^ and a maximum injection time of 50 ms. DIA MS2 spectra were collected at 15,000 resolution (AGC 1 × 10^6^; maximum injection time 27 ms) using 38 staggered isolation windows of 20 m/z and normalized collision energy 28% (27). Data were acquired using Xcalibur software (Thermo Fisher Scientific). The raw MS data and associated analysis parameters deposited to the ProteomeXchange Consortium via the MassIVE partner repository under dataset identifier MSV000100433. Protein group intensities for all fractions (global (G), cytoplasmic (C), and nuclear (N)) and heart chambers (LV, RV, LA, RA) is provided (Supplemental Data).

### MS Data Processing and Analysis

DIA-MS data were processed using DIA-NN v1.8 (28) using our established workflow (27, 29). An additional spectral library of mouse heart nuclear fractions analysed in DIA workflows was generated to enhance spectral coverage (acquired on the same instrument and under closely related LC conditions) (provided in MassIVE). Spectra were searched in library-free mode against the UniProt mouse proteome (UP000000589, 55,086 entries) (30). FASTA-based library generation, deep-learning prediction of MS/MS spectra, retention time, and ion mobility, and match-between-runs were enabled. Searches used Trypsin/P specificity with up to one missed cleavage, precursor charges of 1 to 4, and an m/z range of 300 to 1800. Carbamidomethylation of cysteine was set as a fixed modification, and variable (no variable modification) modifications were kept as default (*i.e.*, N-term M excision, C-carbamidomethylation). All other DIA-NN parameters were at default, including automated mass-accuracy optimization and single-pass neural-network classification. Precursor identifications were filtered at 1% FDR. To ensure high-confidence quantification, protein groups were retained only if quantified in at least 11 of 16 replicates (≥67%) within each fraction. For region-specific nuclear analyses, protein inclusion required valid values in at least three of four biological replicates per region. LFQ intensities were log2-transformed and normalized using variance-stabilizing normalization (VSN), implemented via the normalize_vsn function in the DEP package (31, 32). Missing values were imputed using msImpute v2 with combined MAR low-rank approximation and MNAR left-censored modelling (33). The same workflow was applied to the nuclear-fraction subset.

Proteomic analyses were performed in R (v4.5.1). GO annotations for mouse genes were retrieved using the org.Mm.eg.db and GO.db Bioconductor packages. UpSet UpSet plots were generated using the *UpSetR* package (34). Waterfall plots, bar plots, profile plots, boxplots, Lollipop plot, and Volcano plot were created using *ggplot2* (35). PCA was performed with prcomp (36). Hierarchical clustering was generated using the Heatmap function from *ComplexHeatmap* (37). k-means clustering was performed with kmeans from the *stats* package (38). Gene Ontology and KEGG pathway enrichment analyses were performed using compareCluster from the *clusterProfiler* package and *g:Profiler* (39, 40).

## Results

### Isolation and Characterization of Nucleus From Four Anatomical Regions of Mouse Heart

**Fig. 1:**
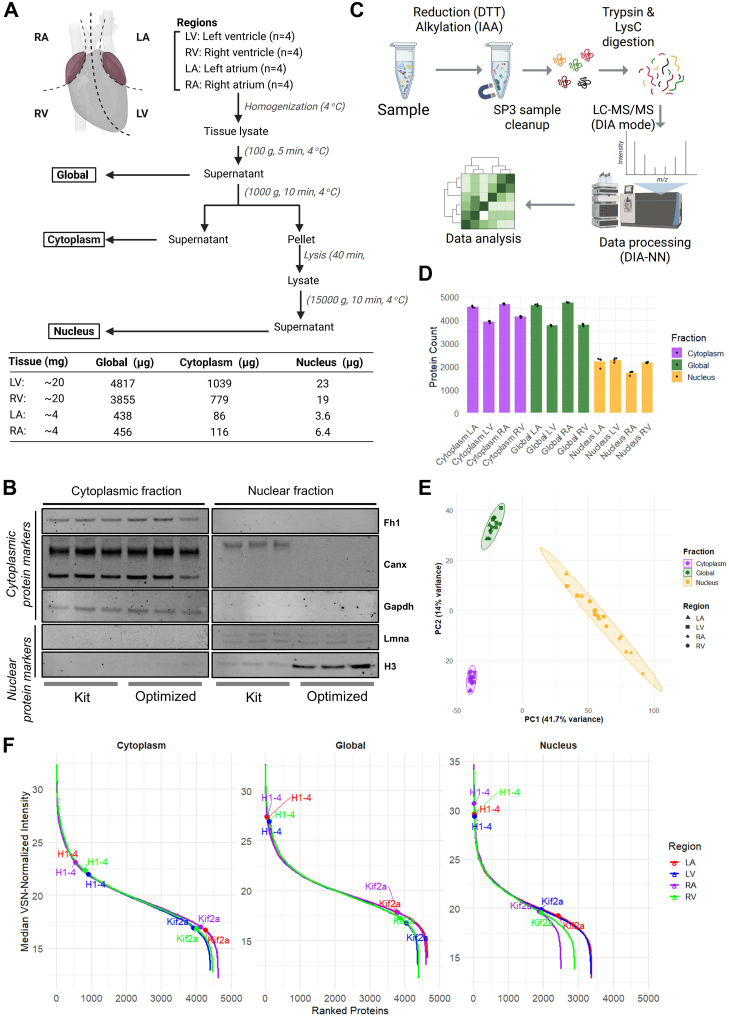
**Experimental and proteomic workflow for nucleocytoplasmic proteome enrichment in the mouse heart.***A*, optimized workflow of nucleocytoplasmic partitioning and nuclear enrichment. Fresh mouse heart regions (N = 4) were homogenized in cell lysis buffer and centrifuged at 100*g*; the supernatant was collected as the Global fraction. The remaining pellet underwent organelle lysis and centrifugation at 1000*g* to yield the Cytoplasmic fraction (supernatant). The final pellet was incubated with nuclear lysis buffer and centrifuged at 15,000*g* to obtain the nuclear fraction. Protein yield in each fraction across regions is shown (Created with BioRender.com). *B*, Western blot validation of nuclear and cytoplasmic markers comparing their intensities across methods and fractions. Cytoplasmic and nuclear fractions were probed for cytosolic/ER markers (Fh1, Canx, Gapdh) and nuclear markers (Lmna, Histone H3), demonstrating reduced cross-contamination and improved nuclear purity with the optimized protocol. *C*, proteomics workflow. Samples underwent reduction (DTT), alkylation (IAA), dual-enzyme digestion (Trypsin & LysC), SP3 cleanup, and LC–MS/MS acquisition in DIA mode, followed by data processing with DIA-NN (Created with BioRender.com). *D*, bar graph showing numbers of protein groups identified after filtering (≥67% valid values across replicates in regions) for Global (G), Cytoplasmic (C) and Nuclear (N) fractions in each heart region. Bars are colored by fraction. *E*, principal component analysis (PCA) of variance-stabilizing normalization (VSN) intensities. Samples cluster primarily by subcellular fraction, with clear separation of Global, Cytoplasmic and Nuclear datasets across regions. *F*, protein rank plots for Cytoplasmic, Global and Nuclear fractions. *Curves* show median VSN-normalized intensity *versus* ranked proteins for each region (LA, RA, LV, RV). Selected nuclear-enriched proteins (Histone H1–4 and Kif2a) are highlighted, illustrating their relative enrichment in the Nuclear fraction.

To isolate nucleus from cardiac tissues, we assessed commercially available nuclear enrichment workflow as described, on the LV region of the mouse heart (Fig. 1, *A* and *B*). Tissue lysate was centrifuged at 15,000*g* to obtain cytoplasm (supernatant) and pellet containing the nucleus. Western blot analysis revealed enrichment of nuclear markers (Histone H3, Lmna) and co-isolation of cytoplasmic organelle marker calnexin (Caxn) in nuclear fraction (kit) (Fig. 1B, Supplemental Fig. S4, *A*–*C*).

To reduce this cytoplasmic carry-over, we subjected the lysate to sequential low-speed centrifugation to first pellet cell debris (100*g*) followed by 1000*g* centrifugation (vs 15,000*g* employed using the kit approach) to obtain nuclear fraction. This resulted in greater enrichment of nuclear protein H3 and concomitant reduction in cytoplasmic marker (Caxn). We next employed this optimized workflow in all four regions of the heart (N = 4 independent samples; regions: LV, RV, LA, RA). We subjected nuclear fraction (N), along with global (G) and cytoplasmic (C) fractions, to label-free mass spectrometry-based proteome analysis (Fig. 1*C* and Supplemental Data). We quantified proteins per region: LA (G = 4728, C = 4675, N = 2368), LV (G = 3855, C = 4034, N = 2403), RA (G = 4902, C = 4764, N = 1816), and RV (G = 3893, C = 4230, N = 2242) (Fig. 1*D* and Supplementary Table S1).

Replicates showed high reproducibility, with a median coefficient of variation (CV) of 14.5% (Supplemental Fig. S1*A* and Supplemental Table S2). Despite their origin from different anatomical regions, principal component analysis (PCA) showed separation of global, cytoplasmic, and nuclear fractions (Fig. 1*E*). Protein-rank plots reveal nuclear proteins (*e.g.*, Histones H1–4 and Kif2a) were among the most abundant proteins in the nuclear fraction relative to the cytoplasmic fraction (Fig. 1*F*). Further protein-level quality control metrics for cytoplasmic, global, and nuclear fractions across cardiac chambers are provided (Supplemental Fig. S1, *B*–*D*).

**Fig. 2:**
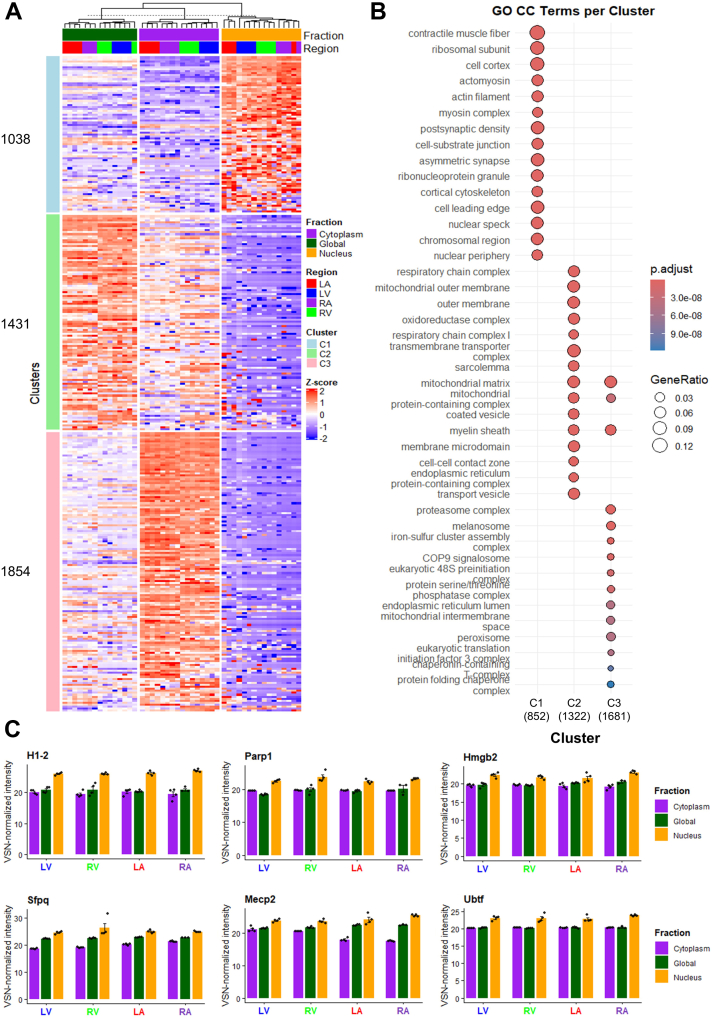
**Comparative analysis of subcellular fractions across mouse heart regions.***A*, heatmap of k-means clustering (k = 3) for significantly differentially abundant proteins (FDR<0.01) across Global, Cytoplasmic and Nuclear fractions in four heart regions (LA, RA, LV, RV). Columns are annotated by fraction and region; rows are grouped into clusters C1–C3, with Z-scored protein intensities shown. *B*, GO cellular component (GO:CC) enrichment analysis for each cluster. Enriched terms were redundancy-reduced using the *simplify* function. Dot size represents GeneRatio; color indicates Benjamini–Hochberg–adjusted *p*-value. Selected representative GO:CC terms are shown for clusters C1–C3. *C*, bar plots show VSN-normalized intensities for representative nuclear proteins (H1-2, Parp1, Hmgb2, Sfpq, Mecp2, Ubtf) across LV, RV, LA, and RA in matched Global, Cytoplasmic, and Nuclear fractions. Bars represent mean ± SEM, with points indicating individual samples. All proteins shown are significantly enriched in the nuclear fraction relative to the cytoplasmic fraction.

Next, we performed limma-based differential abundant analysis of global, cytoplasmic, and nuclear proteomes across heart regions, identifying 4323 significant proteins between fractions (FDR <0.01). Unsupervised k-means clustering (k = 3) revealed three distinct protein clusters (Fig. 2*A* and Supplemental Table S3). Cluster C1 (1038 proteins) associated with the nuclear fraction includes core nuclear hallmark proteins (*e.g.*, H1-(1–3), Hmgb2, Ubtf, Parp1, Sfpq, Mecp2) and enriched in GO terms (FDR <0.01) including nuclear periphery, nuclear speck, chromosomal region, actin filament. In contrast, cluster C3 (1854 proteins), associated with cytoplasmic fraction, included hallmark proteins of cytoplasmic (*e.g.*, Gapdh, Pkm, Ldha), lysosomal (*e.g.*, Tollip, Rab5a, Vps37b), and proteasomal cell compartment (*e.g.*, Psme4, Usp7, Ubqln1). This cluster was enriched in GO terms peroxisome, chaperone complex, proteasome, mitochondrial protein complexes. Further, cluster C2 (1431 proteins), associated with global tissue fraction, was predominantly associated with membrane and organelle-associated proteins (*e.g.*, Atp1a1, Cdh2, Ryr1, Por, Immt) and GO terms sarcolemma, endoplasmic reticulum, mitochondrial outer membrane, cell–cell contact zone (Fig. 2*B* and Supplemental Table S4). Consistent with these assignments, representative cluster C1 nuclear markers showed significantly higher protein abundance in the nuclear fraction than in the cytoplasmic fraction across all regions (Fig. 2*C*).

### Benchmarking Heart Nuclear Proteome Landscape

**Fig. 3:**
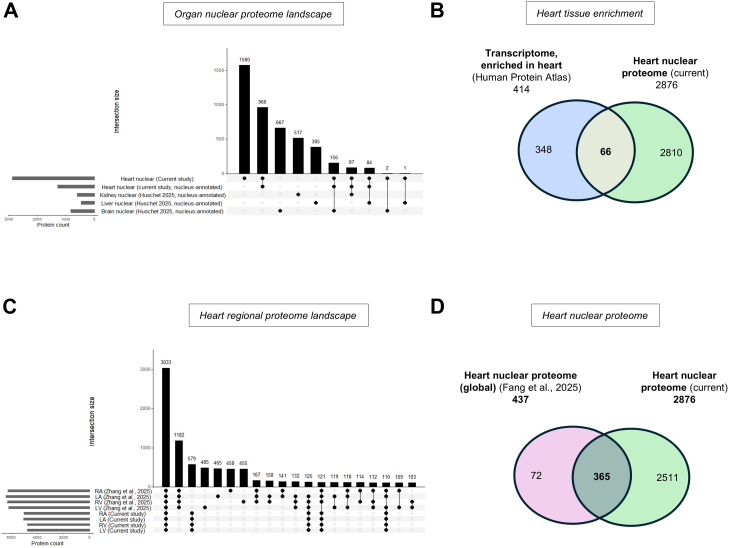
**Benchmarking heart nuclear proteome landscape at heart tissue, heart region, and nuclear heart levels.***A*, UpSet plot comparing heart nuclear proteome (current) with nuclear proteome of brain, liver, and kidney. Comparative analyses further included GO cellular component nuclear-annotated proteins for all mapped proteins, with 966 proteins uniquely identified in current heart nuclear proteome study relative to GO cellular component nucleus-annotated subset of each organ (brain, liver, kidney) nuclear proteome. *B*, Venn comparison of heart tissue enriched analysis (transcript) from Human Protein Atlas (HPA), with UniProt identifiers used in correlative analysis. *C*, UpSet plot comparing mouse chamber-resolved nuclear proteome from mouse *right* atrium (RA), left atrium (LA), *right* ventricle (RV), and *left* ventricle (LV). *D*, Venn comparison of heart nuclear proteome using global density-based fractionation of heart to resolve different subcellular niches of the mouse heart, including the nucleus. Validation against heart nuclear proteome revealed 365 co-identified proteins, 72 of which are identified across all anatomical regions of the mouse heart nuclear proteome.

To benchmark proteome coverage, we compared our dataset across different levels, including heart tissue enrichment networks (41), nuclear isolation from different organs (23) (Fig. 3, *A* and *B*), anatomical region-resolved proteome composition of heart (21) (Fig. 3*C*), and heart nuclear proteome landscape using a global subcellular analysis workflow (19) (Fig. 3*D*).

We demonstrate significant nuclear proteome coverage of the heart in comparison to liver, kidney and brain nuclear proteomes, with 1580 proteins unique to the heart nuclear proteome landscape (Fig. 3*A* and Supplemental Table S16). Here we show 966 nucleus-annotated proteins not identified relative to other organ nuclear proteomes (23), and co-identified proteins with brain (156 proteins), liver (84 proteins), and kidney (87 proteins).

Relative to heart tissue enriched transcripts (41), we highlight 66 proteins in our nuclear proteome analysis from heart, including contractile and cytoskeletal-nuclear connector proteins (Desm, Ppla, Tri55), organization of sarcomeric actin (Lmod2/Lmod3, Telt), cardiomyocyte differentiation (Alpk3), vascular remodeling and cardio homeostasis (Nppa), and anionic signaling (Vdac3) (Fig. 3*B* and Supplemental Table S18). We highlight the significant underrepresented nuclear proteome coverage in these tissue enrichment datasets, reflective of their low abundance, nuclear localization, and association with heart tissue at proteome level.

We further analyzed our nuclear proteome with regional mouse-heart proteome (24), which employed direct global proteome analyses combined with extensive offline peptide fractionation (Supplemental Table S5). In comparison to proteome depth, we observed substantial overlap in protein identifications and an additional 718 proteins identified unique to this current study (Supplemental Fig. S1*E*). Further comparison with regional isolated heart proteomes (21) revealed 579 unique proteins in our nuclear proteome across all regions, and 3033 proteins commonly identified, supporting the resolution of the mouse heart regional proteome (Fig. 3*C* and Supplemental Table S19).

Through systemic subcellular proteome mapping of mouse heart (19), we highlight 365 co-identified proteins in nuclear fraction from mouse heart (Fig. 3*D* and Supplemental Table S17). For these co-identified nuclear protein networks, include enrichment networks include nuclear transport (p-val, 2.09E-02), transcription factor binding (1.89 E−02), tropomyosin binding (4.17E-08), regulation of RNA splicing (8.81E-09), nuclear lumen (7.50E-11), nucleoplasm (7.80E-15), and nuclear body (9.98E-18). These networks provide validation from the heart of nuclear-localizing proteome.

These data show a meaningful and comprehensive construction of the nuclear landscape of different anatomical regions of mouse heart.

### Diversity of Nuclear Proteome Landscape Across Heart Regions

**Fig. 4:**
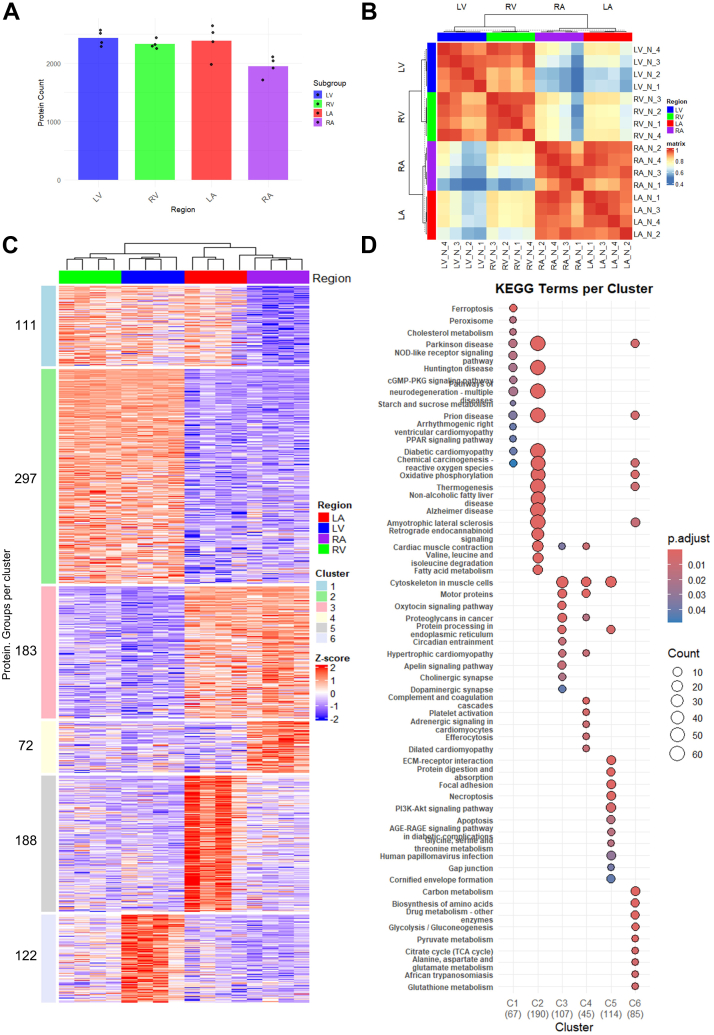
**Regional and functional diversity of the cardiac nuclear proteome.***A*, average protein count in the nuclear fraction after filtering (≥67% valid values in at least one region) across LV, RV, LA, and RA. Bars represent mean values; dots indicate individual biological replicates. *B*, sample-to-sample correlation heatmap of nuclear proteomes showing clustering of biological replicates and separation of atrial (LA/RA) *versus* ventricular (LV/RV) profiles. *C*, heatmap of significantly regulated proteins (n = 973, FDR <0.05) identified by limma ANOVA. *k*-means clustering (*k* = 6) revealed six chamber-specific profiles (C1 = 111, C2 = 297, C3 = 183, C4 = 72, C5 = 188, C6 = 122). Columns are annotated by region (LV, RV, LA, RA); rows are Z-scored protein intensities ordered by cluster. *D*, KEGG pathway enrichment (Benjamini–Hochberg *q* < 0.05) for each cluster. Dot size indicates the number of mapped proteins (Count); color represents adjusted *p*-value. Numbers beneath each cluster indicate KEGG-mapped proteins relative to total cluster size (C1 = 67/111, C2 = 190/297, C3 = 107/183, C4 = 45/72, C5 = 114/188, C6 = 85/122). C1 was enriched for ferroptosis, peroxisome and cholesterol metabolism; C2 for oxidative phosphorylation and cardiac muscle contraction; C3 for cytoskeletal and oxytocin/apelin signaling; C4 for complement and platelet signaling; C5 for ECM–receptor interaction, focal adhesion and PI3K–Akt signaling; and C6 for glycolysis, TCA cycle and oxidative metabolism.

Protein identifications were broadly comparable across nuclear fractions from LV, RV, LA, and RA (Fig. 4*A*). Data showed well-aligned intensity distributions and stabilized variance across samples, with low per-sample CVs indicating high reproducibility in tissue/chamber extraction/dissection, nuclear isolation and proteome analyses (Supplemental Fig. S2, *A*–*F*). Sample-to-sample correlation clustering showed tight grouping of biological replicates and a clear atrial (LA/RA) *versus* ventricular (LV/RV) separation (Fig. 4B), indicating robust region-associated nuclear proteome differences (Supplemental Table S6).

To define chamber-specific features of the nucleus we performed differential abundant analysis on the nuclear fractions of the four heart regions. A one-way limma-based ANOVA across LV, RV, LA, and RA nuclear fractions identified 973 proteins with significant region-dependent abundance differences (FDR <0.05). These 973 represent the total set of significant proteins detected in the nuclear fraction, of which 361 proteins were assigned with nuclear annotation based on GO reference. k-means clustering identified six chamber-associated protein clusters (k = 6; Fig. 4*C*; C1–C6) (Supplemental Table S7).

We observe a striking clustering of ventricle (Cluster 2, 297 proteins) *versus* atrial (Cluster 3, 183 proteins) nuclear proteome. KEGG-based enrichment revealed Cluster 2 (ventricle) associated with nuclear proteins that regulate “oxidative phosphorylation” and “cardiac muscle contraction” (*e.g.*, YY1, Gatad1, Nfix, Mbnl1, Rbm24) pathways, supporting higher metabolic and contractile demand in ventricular myocardium (Fig. 4*D* and Supplemental Tables S8). In contrast, Cluster 3 (atria) networks associated with circadian entrainment, cytoskeletal-regulatory and oxytocin/apelin-signaling components (*e.g.*, Lmna, Camk2d, Itpr1, Gnai1), indicating specialized mechanosignaling and calcium-dependent transcriptional control in atria. These findings of nuclear proteome annotation strongly align with well-established functions of the heart ventricle and atria. Further, we identify Cluster 4 (right-atria dominant feature), associated with complement activation, platelet activation, and adrenergic signaling (*e.g.*, Paip1, Yars1, Ppp1r12a, Apba1), suggesting immune–metabolic adaptation in right atria. Cluster 1 (negative feature of RA) was enriched for ferroptosis, peroxisome organization, and cholesterol metabolism (*e.g.*, Pex14, Pcyt1a, Egln1, Glrx3), highlighting redox and lipid-regulatory programs shared between atrial and ventricular nuclei. In addition, Cluster 5 (left-atria dominant) was associated with ECM–receptor interaction, focal adhesion, PI3K–Akt signaling and apoptosis (*e.g.*, Sun2, H2ac21, Stat5b, Pip4k2c), consistent with nuclear coordination of matrix remodeling and survival signaling in left atria. Further, we identify Cluster 6 (left-ventricle dominant) associated with glycolysis, TCA cycle, and oxidative-metabolism pathways (*e.g.*, Ptbp2, Nme1/2, Hdac6, Senp6), emphasizing metabolic specialization and nuclear coupling to energy-linked processes in the LV.

**Fig. 5:**
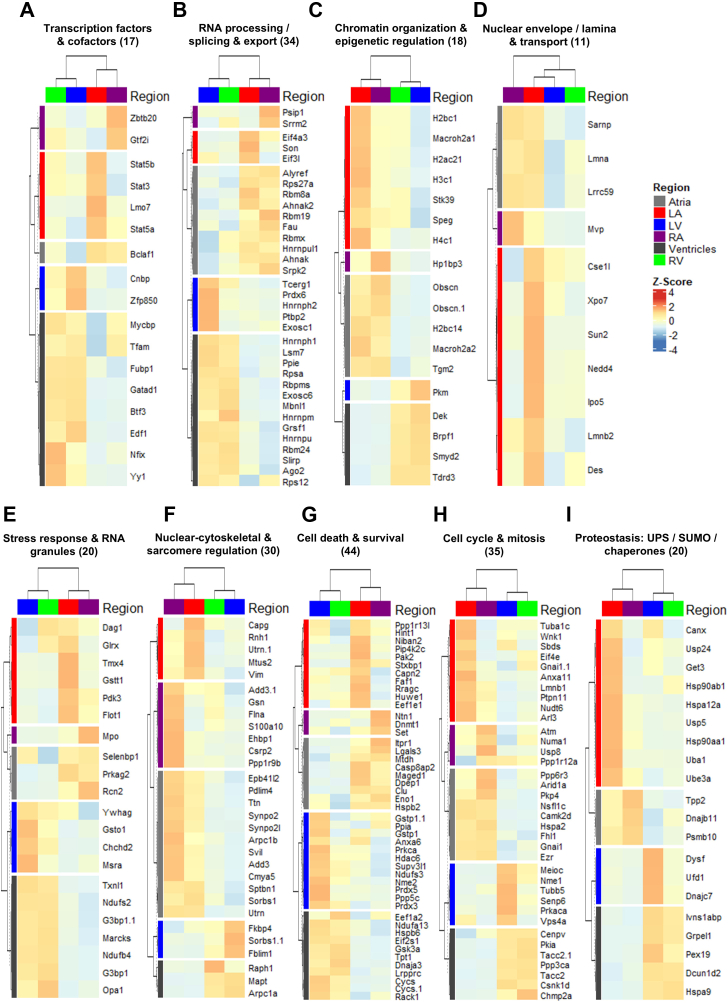
**Hallmark nuclear programs show region-associated signatures across the cardiac nuclear proteome.***A–I*, heatmaps of VSN-normalized, Z-scored abundances for nucleus-annotated proteins selected from the 973 region-dependent nuclear proteins (limma one-way ANOVA across LV, RV, LA, RA; FDR <0.05). Proteins are grouped into functional nuclear programs: *A*, transcription factors and cofactors. *B*, RNA processing/splicing/export. *C*, chromatin organization and epigenetic regulation. *D*, nuclear envelope/lamina and nucleocytoplasmic transport. *E*, stress response and RNA granules. *F*, nuclear–cytoskeletal and sarcomere regulation. *G*, cell death and survival. *H*, cell cycle and mitosis, and *I*, proteostasis (UPS/SUMO/chaperones). Columns represent heart regions (LV, RV, LA, RA; color-coded as indicated) and rows represent individual proteins; values are displayed as Z-scores within each protein. Numbers in parentheses indicate proteins shown per program. Full protein lists are provided in Supplemental Table S9.

We next focused on hallmark features of nuclear landscape which include transcriptional factors and cofactors, RNA processing/splicing & export proteins, chromatin organization & epigenetic regulation, nuclear envelope/lamina & transport, stress response & RNA granules, nuclear-cytoskeletal & sarcomere regulation, cell death & survival, cell cycle & mitosis, and proteostasis: UPS/SUMO/chaperones (Fig. 5, *A*–*I* and Supplemental Table S9). These functional program assignments were manually curated using GO cellular component/biological process annotations and supported by literature evidence. Of the 973 significant differential abundant proteins, the nucleus-annotated subset included 17 proteins identified associated with transcription factors & cofactors, including eight proteins were associated with the ventricle (e.g., Gatad1, Mycbp, Nfix, Tfam), and specific localization of proteins associated with the LA (*e.g.*, Stat3, Stat5a, Stat5b) and LV (*e.g.*, Cnbp, Zfp850) regions, consistent with region-specific cardiac transcriptional programs. In addition, 34 proteins were identified implicated in RNA processing/splicing & export; of these, 14 associated with the ventricle *versus* 10 proteins in atria. These include known RNA processors such as Rbm24 in ventricles (42), and Ahnak-1/2 in atria. We also identify LV specific processors including Ptbp2, which is known to regulate alternative pre-mRNA splicing (43). Among 18 chromatin/epigenetic regulators, several histone and histone-variant proteins (*e.g.*, H2bc1, H2ac21, H3c1, H4c1, Macroh2a1) showed higher abundance in LA. Among 11 nuclear envelope/lamina and transport proteins, atria showed higher Lmna/Lrrc59, while LA was enriched for Cse1l, Xpo7, Ipo5, Sun2, and Lmnb2.

Selected region-enriched candidates were validated by immunofluorescence microscopy for three nuclear-enriched and regionally associated proteins, including H2ac21 and Sun2 in LA and Ptbp2 in LV (Fig. 7, *A*-*I*). The remaining programs, spanning stress/RNA granules, nuclear–cytoskeletal coupling, cell death/survival, cell cycle/mitosis, and proteostasis, displayed distinct region-associated signatures. These findings demonstrate that our systemic framework in nuclear isolation and proteome analyses can maps the anatomical locations of nuclear proteins. Our data show a distinct nuclear proteome across the heart regions, extending chamber-dependent specialization aligned with mechanical load, metabolic demand, and signaling context.

### Core Nuclear Architecture of the Heart

**Fig. 6:**
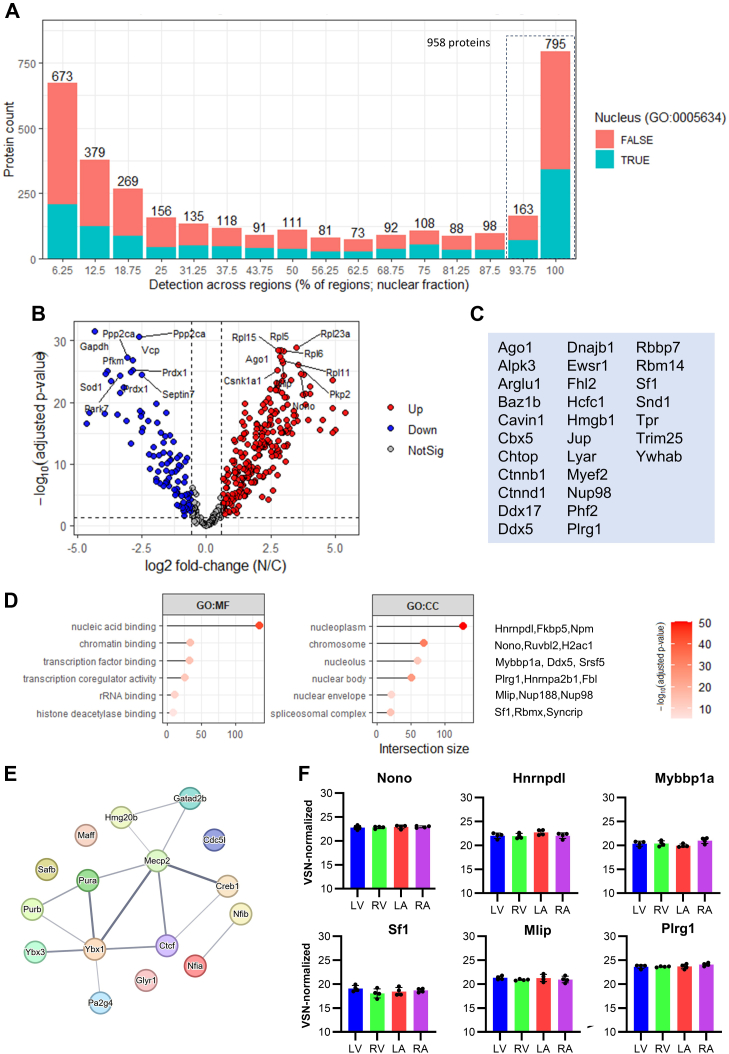
**Definition of a conserved core nuclear architecture across cardiac regions.***A*, frequency distribution of nuclear protein detection across samples, used to define a conserved set. Proteins that were non-significant across regions (ANOVA FDR ≥0.05) and detected in 15/16 or 16/16 nuclear samples were retained as the conserved set (958 proteins). Bars indicate the number of proteins per detection bin; colors denote whether proteins carry GO nucleus annotation (GO:0005634). *B*, volcano plot comparing matched nuclear *versus* cytoplasmic fractions for the nucleus-annotated subset, identifying proteins significantly enriched in the nuclear fraction (FDR <0.05; |fold-change| ≥ 1.5). The resulting 230 proteins (up in nucleus) were defined as the conserved nuclear core. *C*, unique transcription factor (TF) and co-factors identified in mouse heart nuclear core proteome relative to mouse heart tissue TFs and DNA-binding proteins network (29). *D*, GO-enrichment analysis of the conserved nuclear core. *Left*: molecular function (GO:MF) terms; *right*: cellular component (GO:CC) terms, highlighting canonical nuclear compartments and functions. *E*, protein–protein interaction network of conserved transcription factors within the nuclear core, visualized as an interaction map. *F*, representative proteins from major GO:CC categories (Nono, Hnrnpdl, Mybbp1a, Sf1, Mlip, Plrg1) show consistent VSN-normalized abundance across LV, RV, LA, and RA, supporting a stable, shared nuclear architecture. Bars represent mean ± SEM with points indicating individual samples.

Of the 2876 proteins in the cardiac nuclear fraction, 1903 showed no significant abundance differences across regions (FDR ≥0.05). To define a conserved nuclear core, we further required robust detection across heart chambers, retaining proteins detected in 15/16 samples/regions (>90%) and 16/16 (100%) nuclear samples across all regions and replicates, yielding 958 proteins (Fig. 6*A* and Supplemental Table S10). Of these, 400 proteins were associated with GO nuclear annotation (GO:0005634).

To enrich nucleus-specific constituents, we compared matched nuclear and cytoplasmic fractions within this nucleus-annotated subset. We identified 230 proteins significantly enriched in the nuclear fraction (FDR <0.05; fold-change ≥1.5), defined as the conserved nuclear core (Fig. 6*B* and Supplemental Table S11). Interestingly, we report 78/230 proteins as transcription factors and associated co-factors (44) in this conserved nuclear core network (Supplemental Table S11), including 29 proteins (e.g., Alpk3, Rbm14, Arglu1, Hmgb1, Myef2, Sf1) uniquely identified in this study compared to mouse heart tissue TFs and DNA-binding proteins network (45) (Fig. 6*C*). GO enrichment of this conserved nuclear core highlighted canonical nuclear compartments, including nucleoplasm (*e.g.*, Hnrnpdl, Fkbp5, and Npm), chromosome/chromatin-associated proteins (*e.g.*, Nono, Ruvbl2, H2ac1), nucleolus (*e.g.*, Mybbp1a, Ddx5, Tcof1), and nuclear envelope/pore components (*e.g.*, Mlip, Nup188, Nup98), alongside molecular functions related to nucleic-acid/chromatin binding and transcriptional regulation (Fig. 6*D* and Supplemental Table S12). Within this nuclear core, we identify 16 conserved transcription factors based on abundance across regions, including Mecp2, Ctcf, Creb1, Nfia/b, and Hmg20b. This protein interaction network highlights the central, highly connected protein features including various critical nuclear factors of cardiac development, structure and autonomic control (Fig. 6*E*). Representative proteins from major GO:CC categories (Nono, Hnrnpdl, Mybbp1a, Sf1, Mlip, and Plrg1) showed consistent abundance across regions (Fig. 6*F*), supporting a conserved abundance in nuclear architecture.

Comparative analyses of this conserved core nuclear cardiac proteome reveal 69/230 nuclear annotated proteins associated identified relative to brain, liver and kidney nuclear protein signatures (23). Together, these results define a conserved nuclear core that remains stable across LA, RA, LV, and RV.

### Validation of Region-Specific Nuclear Proteins by Immunofluorescence

**Fig. 7:**
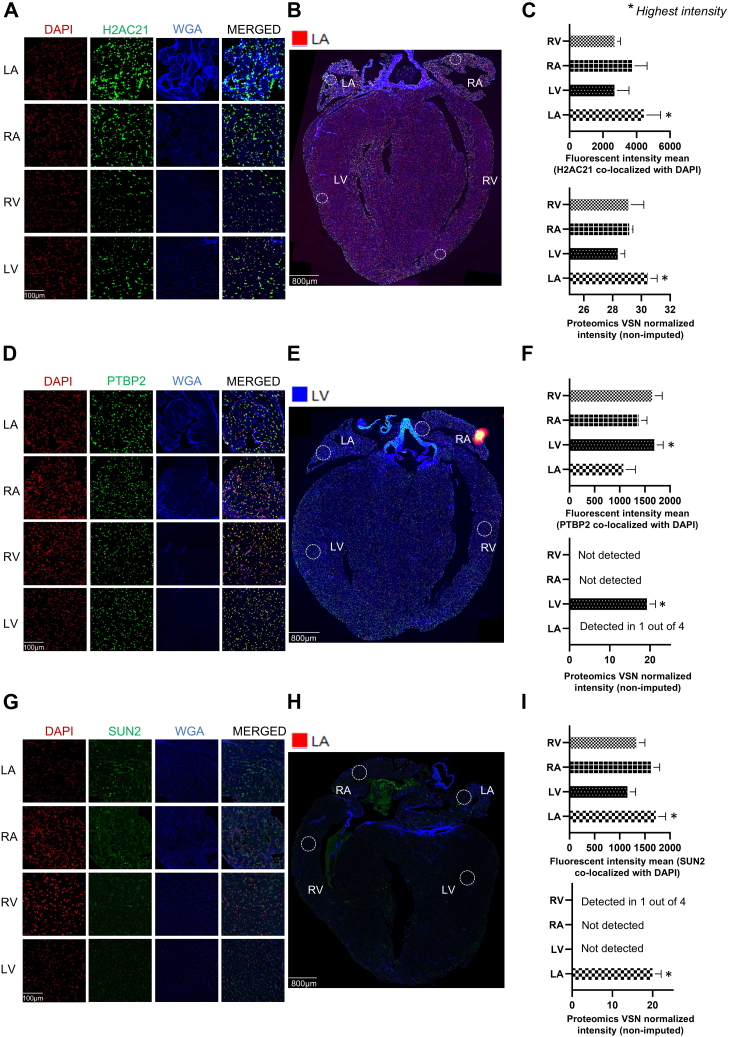
**Validation of region-dominant nuclear proteins by immunofluorescence.***A*–*C*, H2AC21, *D–F*, PTBP2, and *G–I*, SUN2. Nuclei are labeled with DAPI (*red*), cell membranes with WGA (*blue*), and target proteins are shown in *green*. *A*, *D*, and *G*, representative high-magnification images from LA, RA, RV, and LV showing nuclear-associated localization patterns. *B, E*, and *H*, whole-heart sections indicating the anatomical regions used for high-magnification imaging (*dashed circles*). *C*, *F*, and *I*, quantification of region-specific enrichment. Upper plots show mean nuclear IF intensity (fluorescence co-localized with DAPI; n = 3 hearts, three fields per chamber, mean ± SEM). *Lower plots* show corresponding VSN-normalized log_2_ proteomic intensities from nuclear fractions (non-imputed; n = 4 hearts, independent group), summarized by region (mean ± SEM). Asterisks mark the chamber with the highest IF signal. “Not detected” and “Detected in one out of four” indicate proteins not quantified in the proteomic dataset or quantified in only one biological replicate, respectively.

To validate region-dominant nuclear signatures identified by MS profiling, three representative nuclear proteins (H2ac21, Ptbp2, and Sun2) were examined by immunofluorescence (IF) across anatomical regions of mouse heart (Fig. 7, *A*–*I*; N = 3 hearts) and compared with regional proteome abundance from MS (N = 4 hearts). Consistent with proteomic clustering (Fig. 4), H2ac21 (C5; left-atrial dominant) displayed a striking nuclear association in LA, with reduced signal in ventricles and RA (Fig. 7, *A*–*C*). Further, Ptbp2 (C6; left-ventricular dominant) displayed the highest nuclear intensity in LV, supporting the association of MS profiling, with RA and RV largely below detection by DIA-MS (Fig. 7, *D*–*F*). We further demonstrate expression of Sun2 (C5; left-atrial dominant), a nuclear-envelope protein linked to ECM–receptor and PI3K–Akt signaling, localized to the nuclear rim of atrial cardiomyocytes with maximal expression in LA (Fig. 7, *G*–*I*). H2ac21 showed compact intranuclear distribution, Ptbp2 localized to nuclear foci, and Sun2 outlined the nuclear periphery, consistent with regional differences in nuclear organization. IF patterns corroborated region-dominant trends inferred from DIA-MS, supporting tissue-level localization of key chamber-associated nuclear programs (Fig. 7, Supplemental Fig. S3, *A*–*D*, and Supplemental Table S13–S15). Minor differences between platforms likely reflect differences in sampling scale and independent cohorts.

## Discussion

In this study, we define the cardiac nuclear proteome across different anatomical regions of the heart. We map the core nuclear architecture and region-dominant nuclear programs in the native/fresh mouse heart and validate their regional localization using immunofluorescence microscopy on heart tissue. This comprehensive label-free, strategy to isolate and identify the nucleus (similarly with the global and cytoplasmic fractions) provides distinct clusters of proteins comprising the heart and defined anatomical regions of the ventricle and atria, and a systemic insight into the diversity of the nuclear cardiac landscape.

We establish an optimized nuclear-enrichment workflow that enables a refined definition of the cardiac nuclear subproteome. Sequential fractionation with additional centrifugation steps improved recovery of chromatin-associated and regulatory proteins while reducing detectable contamination from abundant cytoskeletal and mitochondrial components. Enrichment of canonical nuclear markers and reduced cytosolic/ER signal indicate that the dataset is enriched for nuclear constituents. Concurrent analyses of global and cytoplasmic fractions support nuclear specificity and contextualize the nuclear proteome within the broader cardiac proteome. Building on prior multi-chamber cardiac proteomic atlases (1, 24), we provide a region-resolved view of the cardiac nuclear proteome and map how transcriptional, chromatin, and nuclear-envelope machinery is organized across chambers.

As a dynamic organ, the heart and detailing the nuclear proteome has largely been challenged by traditional proteomic global approaches due to the unique structural complexity and cellular diversity of the cardiac environment. Contractile protein networks associated with the machinery of the heart, the extracellular matrix, and organelle diversity of the heart present challenges in isolation of the nuclear network and dynamic range, including understanding low-abundance proteins (19). Through comparative nuclear annotated proteomics, we highlight a network of nuclear proteins associated with the heart (966 proteins, Fig. 3*A*), relative to brain, liver, and kidney tissue-specific nuclear protein signatures (23). These heart-specific regulatory/structural factors include various TFs conserved across regions of the heart that that play cell type–specific roles, including Mecp2 (master regulator of heart formation and contractile function), Ctcf (DNA organization, key regulator of mitochondrial biogenesis), and Hmg20b (chromatin-binding protein, cytokinesis regulator). Indeed, when compared to a recent characterization of tissue-type restricted TFs and DNA binding co-factors (45), we obtained 78 TFs overall in the heart nuclear proteome and 29 unique factors associated with the heart—including transcription coregulator activity, nuclear receptor coactivation, chromatin remodeling, submembrane organization, and splicing factor binding.

Further, the nuclear dataset supports a model in which a conserved nuclear backbone is overlaid by region-dominant programs that reflect the discrete roles of cardiac chambers. Across nuclear fractions, many proteins showed no significant chamber-dependent differences and were consistently detected across samples, irrespective of their origin, indicating a broadly shared nuclear cardiac proteome. By restricting this set to nucleus-annotated proteins and nuclear enrichment stringency (nucleus *versus* cytoplasm), we defined a conserved nuclear core of 230 proteins spanning canonical components of the nucleoplasm, chromatin/chromosome (Nono, Ruvbl2, H2ac1) (46, 47, 48), nucleolus (Mybbp1a, Ddx5, Srsf5) (49, 50, 51), and nuclear envelope/pore (Mlip, Nup188, Nup98) (52, 53, 54). Notably, this core includes a connected network of conserved transcription factors (*e.g.*, Creb1, Mef2c, Ctcf, Hmg20b, Nfia/b) (55, 56, 57, 58, 59), supporting the concept that chamber specialization is built on a shared regulatory scaffold, with region-specific programs layered rather than arising from entirely distinct nuclear systems (60, 61). Together, these data transition beyond descriptive regional proteomics by defining a chamber-spanning nuclear architecture with a stable core and layered, region-specific programs.

This study also identified ventricle associated nuclear network from the heart, that align with the ventricular programs in metabolic and contractile regulation (62). In ventricles-dominant protein features (LV + RV), higher nuclear abundance of regulators such as Yy1 (63) and Tfam (64), together with RNA-binding/splicing factors including Mbnl1 (65, 66) and Rbm24 (67), were observed. Such insights are consistent with nuclear control of transcriptional and RNA-processing modules that support the high energetic and contractile demand of ventricular myocardium. The LV-dominant set further advance this profile, enrichment of Ptbp2 (43, 68) together with chromatin- and redox-linked factors (*e.g.*, Hdac6, Nme1/2, and Prdx6) (69, 70, 71, 72), suggests that left-ventricular nuclei ascribe connected features of RNA processing, epigenetic regulation, and stress-responsive proteostasis in the systemic ventricle, which sustains the highest cardiac workload. Consistent with this, prior studies have linked Nme2 (NDPK-B) deficiency to LV hypertrophy and diastolic dysfunction, with increased fibrotic signaling and Ca^2+^ handling, in part via endothelial hexosamine biosynthesis and O-GlcNAc dysregulation (73, 74).

Atrial region-dominant programs show a different balance of nuclear functions, with stronger representation of nuclear-envelope and signaling-linked modules. The atria-dominant set is marked by nuclear envelope/lamina components such as Lmna (75). This region also includes signaling- and stress-linked proteins, including Camk2d (76, 77) and Hspb2 (78, 79). Together, this pattern suggests that atrial nuclei integrate nuclear-envelope/LINC architecture with nucleo–cytoskeletal coupling (80). Such insights are consistent with enhanced Ca^2+^-dependent signaling and stress adaptation in atria (76, 79). Within this framework, RA-dominant profiles are enriched for chromatin and cytoskeletal regulators (*e.g.*, Dnmt1, Hmga1, Flna) (81, 82, 83, 84, 85, 86). This pattern is consistent with coupling between epigenetic control and actin-linked mechanics. In contrast, the LA-dominant program is marked by Sun2 (87), the histone variant H2ac21 (88, 89), and Stat3/Stat5b (90, 91). Such associations in nuclear factors align with pathways that link nuclear-envelope and chromatin organization to survival signaling and structural remodeling. Supporting this interpretation, cardiomyocyte-specific deletion of Stat3 produces an HFpEF-like phenotype with hypertrophy, fibrosis, and diastolic dysfunction (92), and disruption of the Nrg-1/Erbb4–Stat5b signaling has been associated with impaired cardiomyocyte growth and cardiac function (91). Together, these patterns and distinct clusters suggest that chamber specialization is achieved largely by differential emphasis on shared nuclear programs, rather than entirely distinct nuclear components.

Given the key data provided within for nuclear protein interactions and localization, we sought to validate various protein features assigned based on proteome enrichment across different anatomical regions of the heart. Indeed, spatial imaging analysis of the heart supported isolation/proteome analysis framework. H2ac21 and Sun2, which selected from left-atrial–dominant clusters, showed strong nuclear and perinuclear signal in LA. In contract, Ptbp2, drawn from the LV-dominant set, showed the strongest nuclear signal in LV. Overall, these chamber-specific staining patterns qualitatively matched the regional trends in label-free MS data, suggesting that the nuclear proteomics signatures are preserved in nuclei within intact atrial and ventricular tissue.

In summary, our results highlight the diversity and conservation in nuclear proteome in the heart. The myocardium shares a conserved nuclear core, but each chamber shifts the balance of nuclear programs. These differences likely reflect chamber-specific mechanical load, metabolic demand, and signaling context. By combining an optimized nuclear-enrichment workflow with in-depth nuclear proteome profiling, region-resolved pathway analysis, and tissue-level spatial IF validation, we built a chamber-resolved reference map of the nuclear proteome in the healthy heart. This resource connects nuclear organization to atrial and ventricular biology, provides a baseline for studying nuclear remodeling, new understanding into cardiac transcriptional networks and potential candidates for tissue engineering and regenerative medicine research.

## Data Availability

Data generated or analyzed during this study are included in this published article (and its supplementary information files) or available from Data Repositories. MS-based proteomics data, including in depth spectral library generation (for mouse nuclear coverage) is deposited to the ProteomeXchange Consortium via the MassIVE partner repository and available via MassIVE with identifier MSV000100433. Protein group intensities for all fractions (global (G), cytoplasmic (C), and nuclear (N)) and heart chambers (LV, RV, LA, RA) is provided (Supplementary Data). Hierarchical clustering was performed in R using Euclidian distance and average linkage clustering, with missing values imputed at z-score 0. R was also used for data visualization.

## Supplemental data

This article contains supplemental data. Supplementary Figs. S1–S4, Supplementary Tables S1–S19, Supplementary Legends, and Supplementary Figure and Table (19, 21).

## Conflict of interest

The authors declare the following financial interests/personal relationships which may be considered as potential competing interests: All authors declare no conflict of interest. DWG is senior editor for Proteomics (Systems Biology) and Journal of Extracellular Vesicles. His association with these journals did not impact the editorial review or the decision to publish this article.

## Supplemental Data

Supplemental data

Sup_updated accordig to the new figure and updates
