# Supplementary material for: Nuclear Proteome Map of Mouse Heart Chambers
Source: Mol Cell Proteomics. 2026 May 14;25(6):101585. doi: 10.1016/j.mcpro.2026.101585 (PMC13272532; doi:10.1016/j.mcpro.2026.101585)

Supp Figure S1

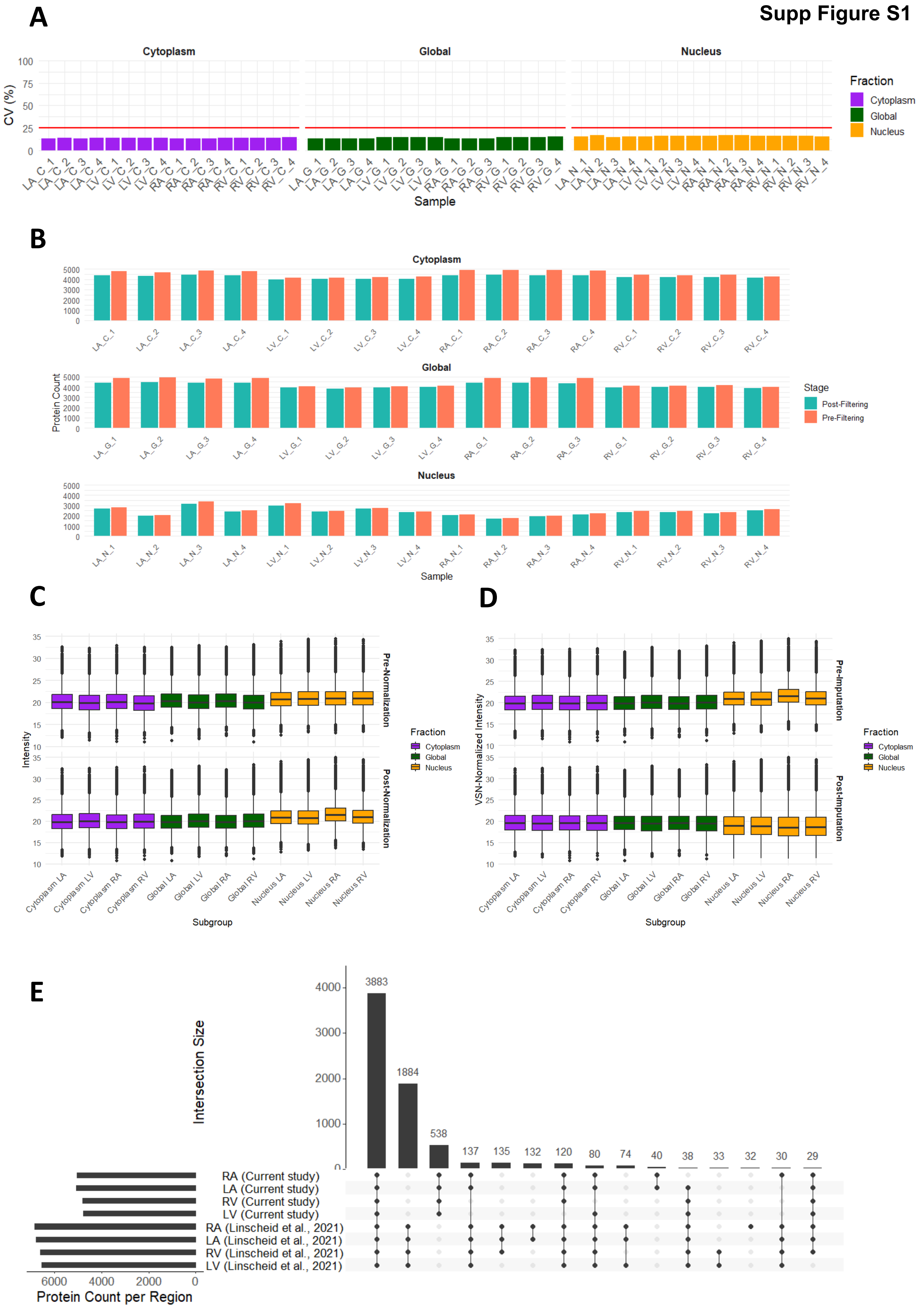

Supp Figure S2

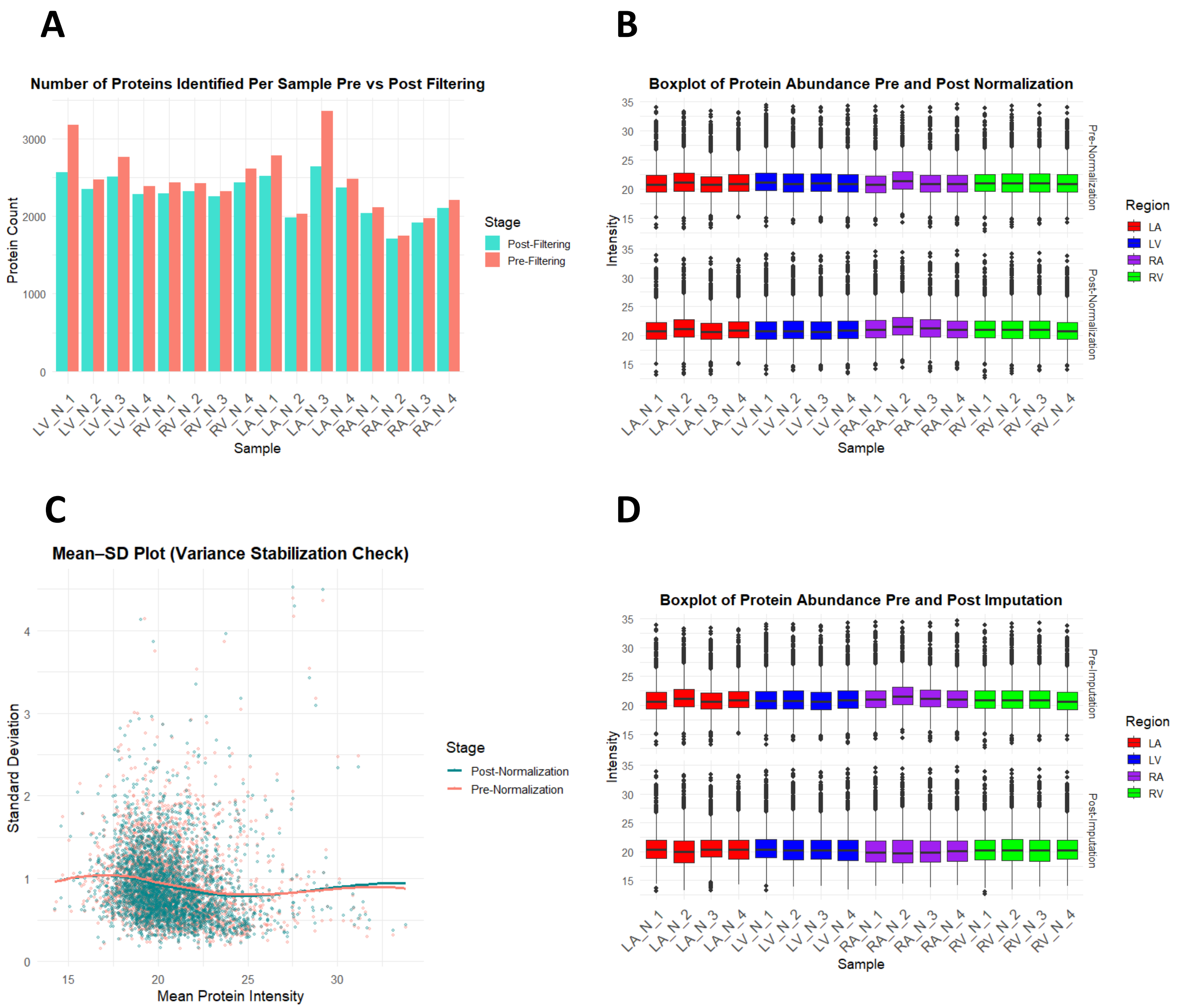

Supp Figure S2

E

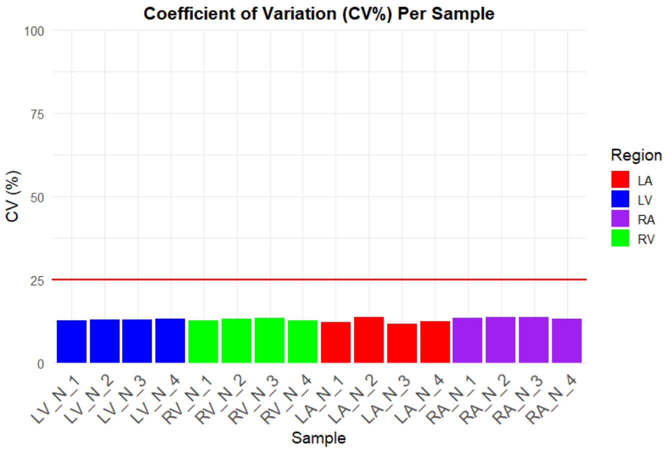

F

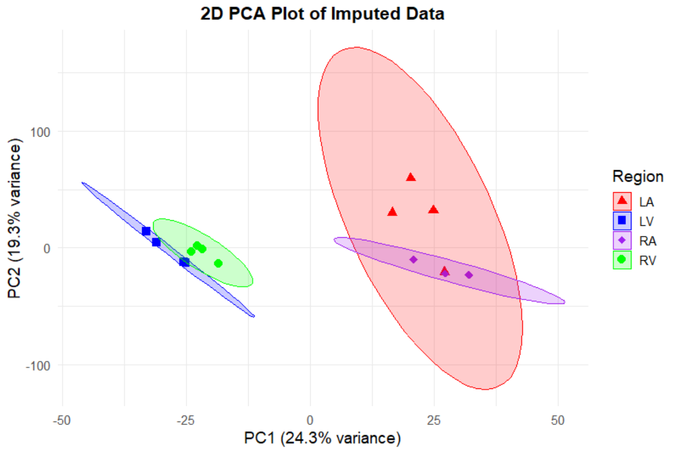

Supp Figure S3

A

Negative background

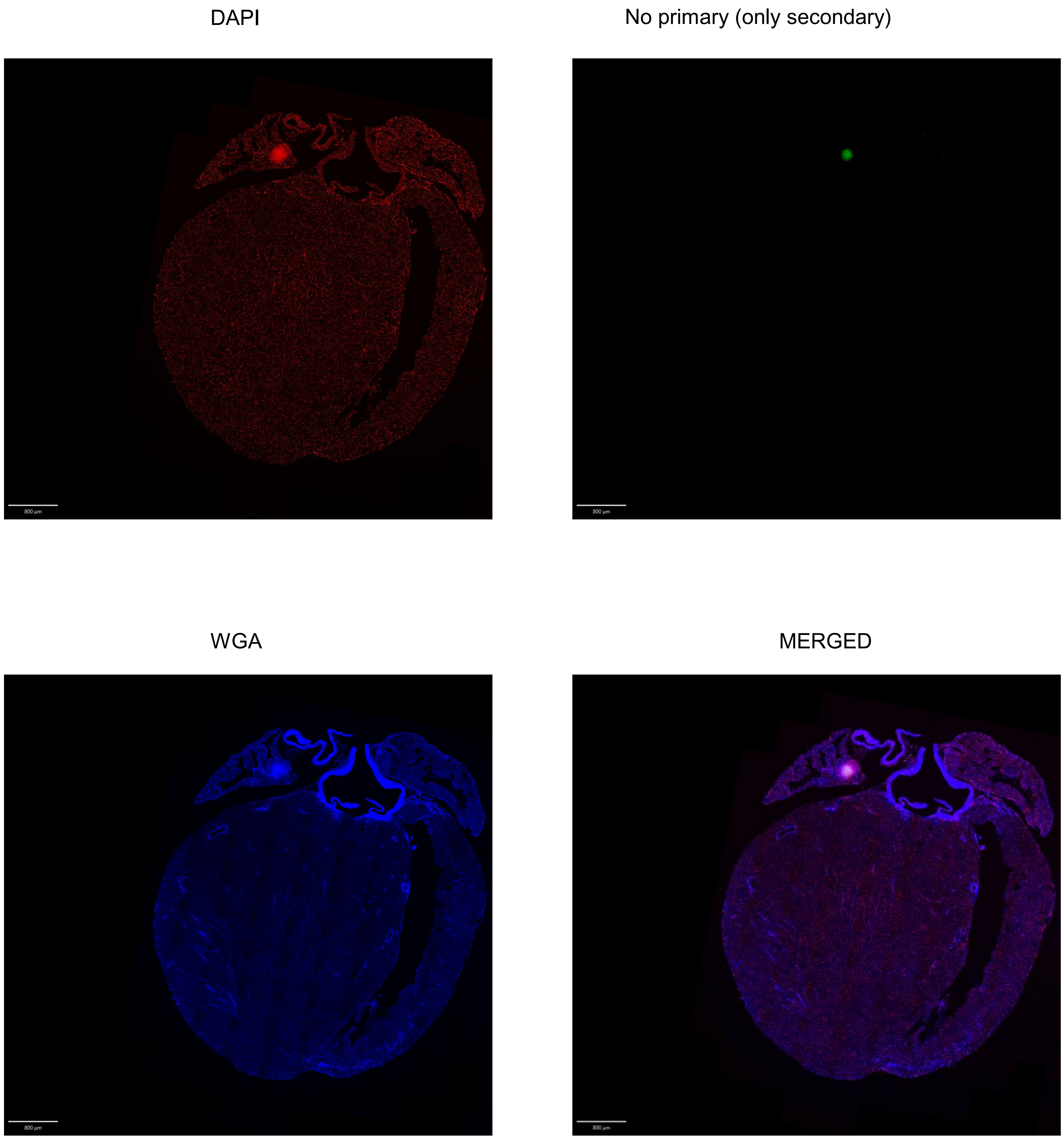

B

H2ac21

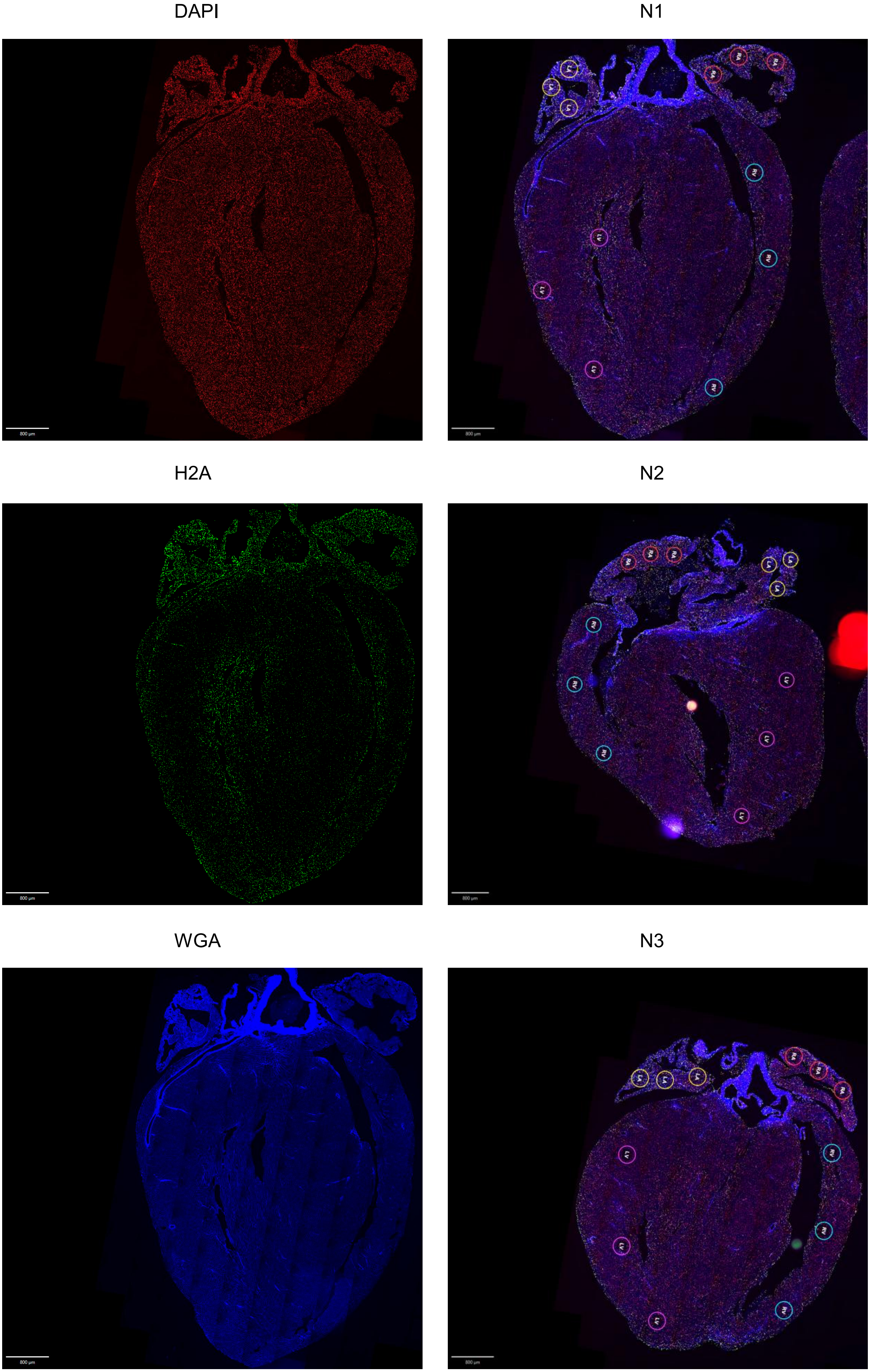

Supp Figure S3

C

Ptbp2

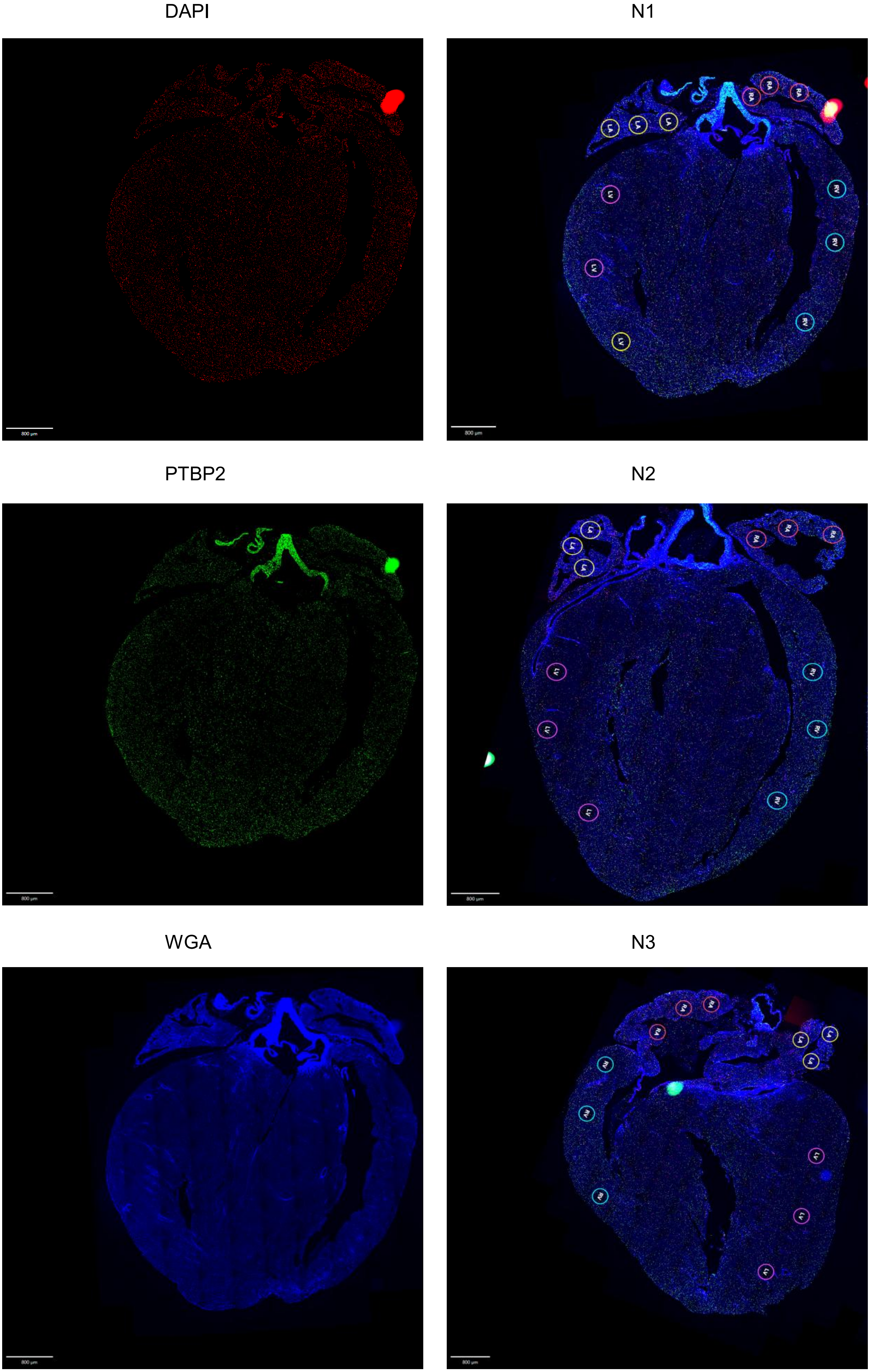

D

Sun2

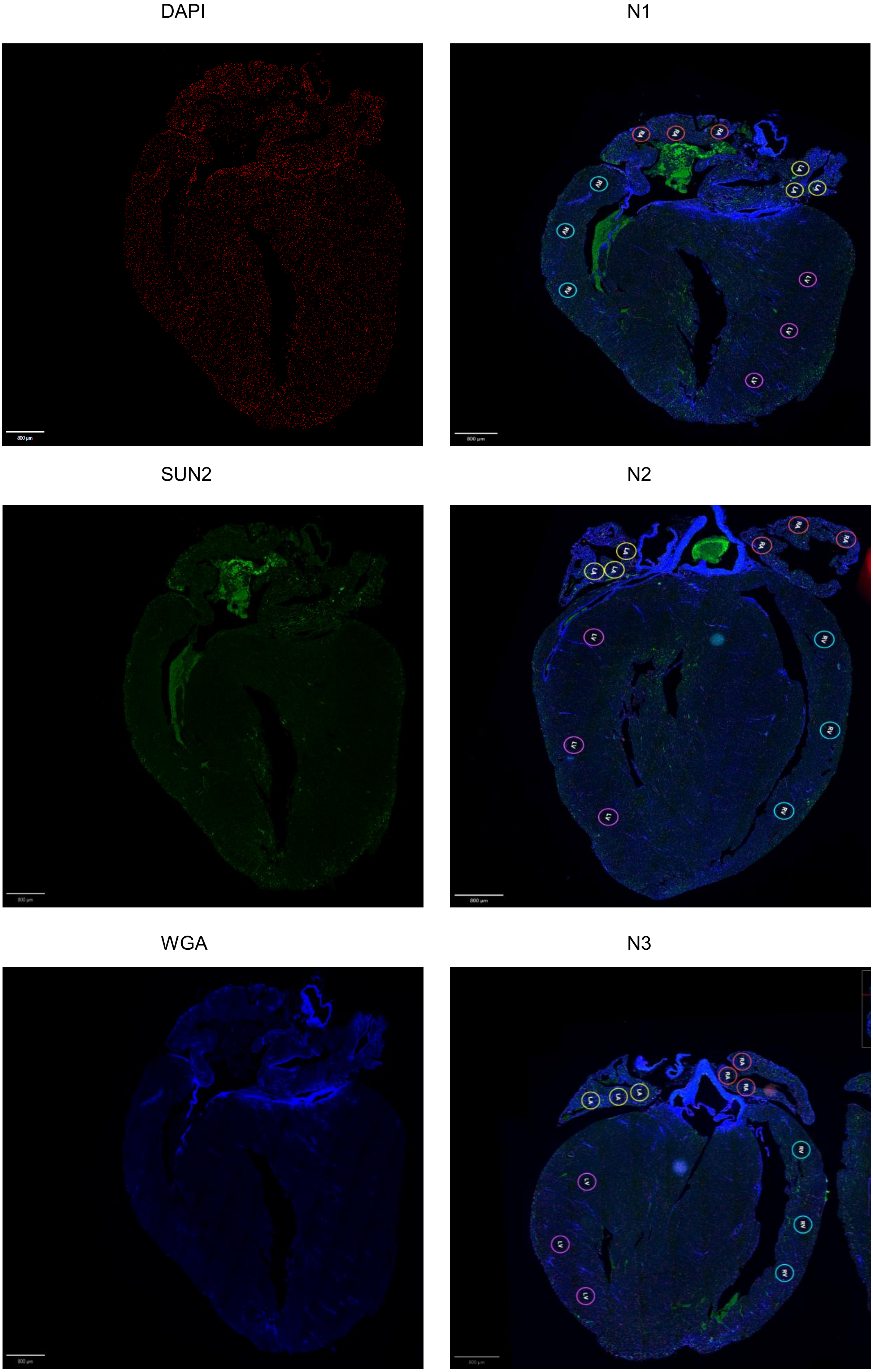

Supp Figure S4

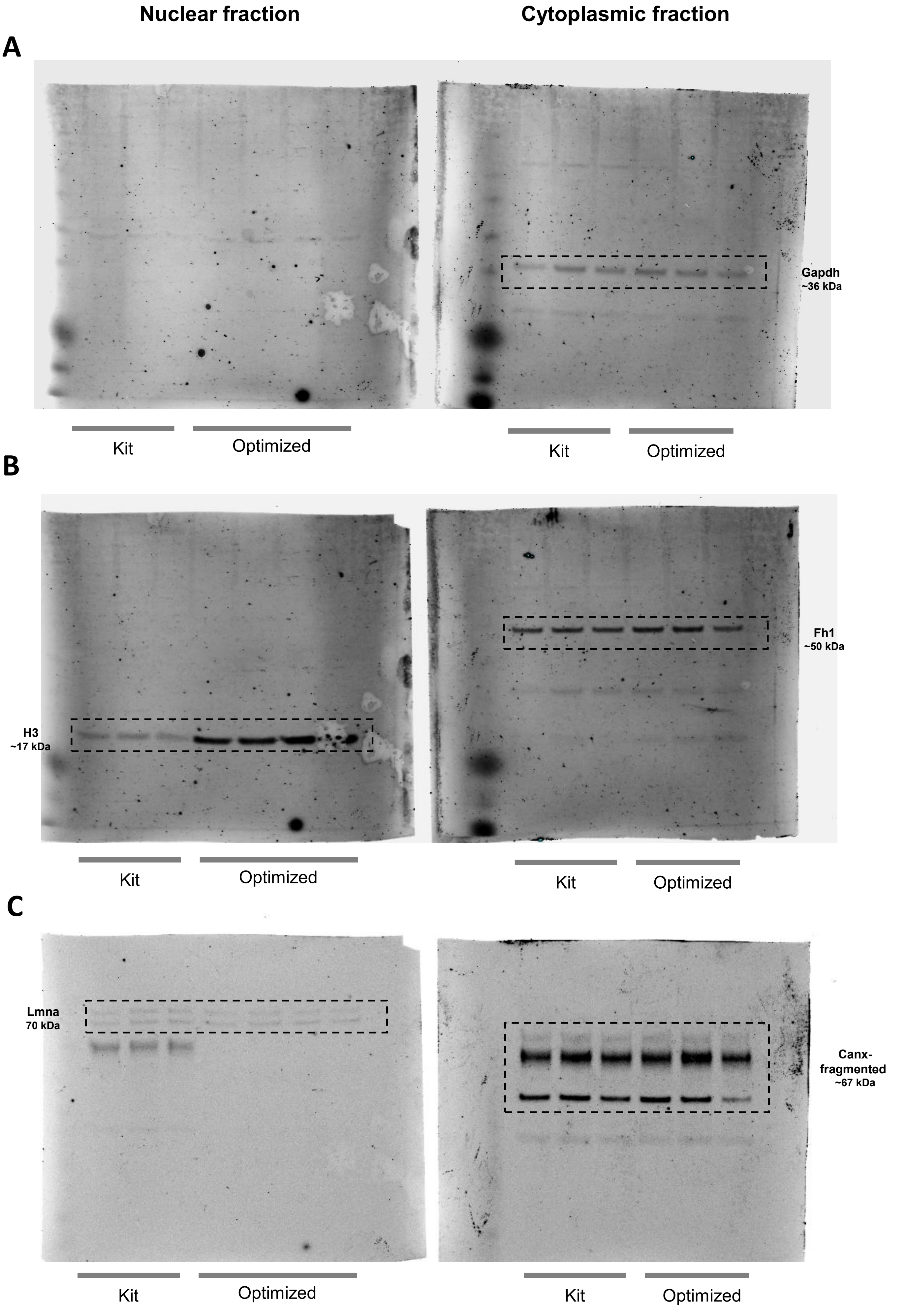

Supplement: Supplemental data [file mmc1.pdf]
